# The utility of circulating LHCGR as a predictor of Down's syndrome in early pregnancy

**DOI:** 10.1186/1471-2393-14-197

**Published:** 2014-06-06

**Authors:** Anne E Chambers, Walter E Mills, Imma Mercadé, Francesca Crovetto, Fatima Crispi, Laia Rodriguez-Revenga Bodi, Michael Pugia, Aurea Mira, Luis Lasalvia, Subhasis Banerjee, Elena Casals, Eduard Gratacos

**Affiliations:** 1 Origin Biomarkers, Biocity Scotland, Bo’Ness Road, Newhouse, Lanarkshire ML1 5UH, UK; 2 Bioquímica i Genètica Molecular, Hospital Clinic Barcelona, c/ Villarroel 170, Barcelona 08036, Spain; 3 Clinical Research, Department of Maternal-Fetal Medicine, ICGON, Fetal and Perinatal Medicine Research Group, IDIBAPS, Hospital Clinic - Universitat de Barcelona, Sabino de Arana 1- Helios III, Barcelona 08028, Spain; 4 Biochemistry and Molecular Genetics Department, Hospital Clínic, Barcelona, Spain; 5 Siemens Healthcare Diagnostics, 3400 Middlebury Street, Elkhart, IN 46516, USA; 6 Center for Biomedical Diagnosis, Hospital Clínic, Barcelona, Spain; 7 Siemens Healthcare Diagnostics, New York, NY, USA

**Keywords:** hCG Receptor, sLHCGR, hCG-LHCGR, ELISA, Down’s syndrome, Prenatal diagnosis, Screening test, Noninvasive prenatal test (NIPT), Early pregnancy

## Abstract

**Background:**

Previous studies showed that soluble LHCGR/hCG-sLHCGR concentrations in serum or plasma combined with PAPP-A and free βhCG significantly increased the sensitivity of Down’s syndrome screen at early pregnancy without altering the false positive rate. The goal of the present study was to further examine the role of sLHCGR forms as combinatorial markers and to investigate whether sLHCGR could serve as an independent biomarker for Down’s syndrome in first trimester pregnancy screens.

**Methods:**

The PAPP-A, free βhCG, and hCG-sLHCGR concentrations together with nuchal translucency (NT) were measured in 40 Down’s and 300 control pregnancies. The sLHCGR concentration was analysed in 40 Down’s and 206 control pregnancies.

**Results:**

The hCG-LHCGR in combination with PAPP-A and free βhCG increased the detection rate (DR) by 35% without altering the false positive rate (FPR). The sLHCGR: hCG-sLHCGR ratio alone detected 80% of Down’s pregnancies in first trimester screening, with a false positive rate of 0.5%.

**Conclusions:**

While measurement of sLHCGR forms in combination with PAPP-A and free βhCG significantly increases the detection rate of Down’s syndrome at first trimester, the ratio of sLHCGR: hCG-sLHCGR acts as an independent marker with a detection rate that is significantly higher than the existing biochemical markers individually for prenatal first trimester screening of Down’s syndrome.

## Background

Down’s syndrome (DS) occurs in about one in 800 pregnancies and live births from affected pregnancies lead to serious post-natal developmental disabilities. The prevalence of such a high frequency of trisomy 21 and other fetal chromosomal aneuploidies led to the introduction of a two-step clinical evaluation: a) non-invasive prenatal screening by quantitative biochemical analysis of maternal blood samples together with fetal ultrasonographic measurements prior to b) invasive and definitive diagnosis by genetic analysis of chorionic villous samples (CVS) and amniotic cells. The highest sensitivity detection rate (DR) of the current first trimester prenatal screening (biophysical and biochemical parameters) is claimed to be between 75% and 90% with a false positive rate (FPR) of 3-5% [1,2]. The recent development of extensive sequencing of fetal DNA extracted from maternal plasma is safer for the pregnancy and outperforms the traditional prenatal screening methods with respect to the sensitivity and specificity of Down’s syndrome screen [3-5].

The human chorionic gonadotropin (hCG) together with its cognate receptor (LH/hCG-R or LHCGR) are ‘master-regulators’ of embryo implantation and pregnancy maintenance. The placental expression of mature LHCGR is severely down-regulated in trisomy 21 pregnancies [6,7]. The functional state of hCG, however, may also be regulated by the circulating soluble LHCGR (sLHCGR). We have demonstrated that maternal serum concentrations of both sLHCGR and hCG-sLHCGR at the first trimester of Down’s pregnancy were outside the normal range, being either undetectable (very low) or extremely high [8].

Our previously published data suggested that simple measurement of hCG-sLHCGR and sLHCGR did not have utility as independent biomarkers for Down’s syndrome, but that they did have utility for the increased detection of Down’s syndrome at first trimester when used in combination with PAPP-A, free βhCG and other biophysical markers [8]. In this report we have taken a different approach towards the analysis of hCG-sLHCGR and sLHCGR measurements in order to further explore their utility as first trimester stand-alone markers for Down’s syndrome.

## Methods

This case‒control retrospective study was on a general population presenting as part of the universal prenatal first trimester screening program for aneuploides (9^+0^-13^+6^ weeks of gestation) at the Department of Maternal‒Fetal Medicine, Hospital Clinic Barcelona. This study approach was approved by the Scientific Ethical Committee of the Hospital Clinic Barcelona, Universitat de Barcelona. All women participating in this study gave written informed consent during first trimester Down's syndrome screening.The samples are derived from retrospective studies at Barcelona Clinic. The control samples were collected on the same day as Down’s (but were without detectable chromosomal abnormalities) and the samples were stored at-80°C under same conditions as the control samples.

Analysis of the serum biomarkers and nuchal translucency (NT) scan were conducted on 12,204 pregnant women of all maternal ages to obtain 40 Down’s serum samples.

**Figure 1:**
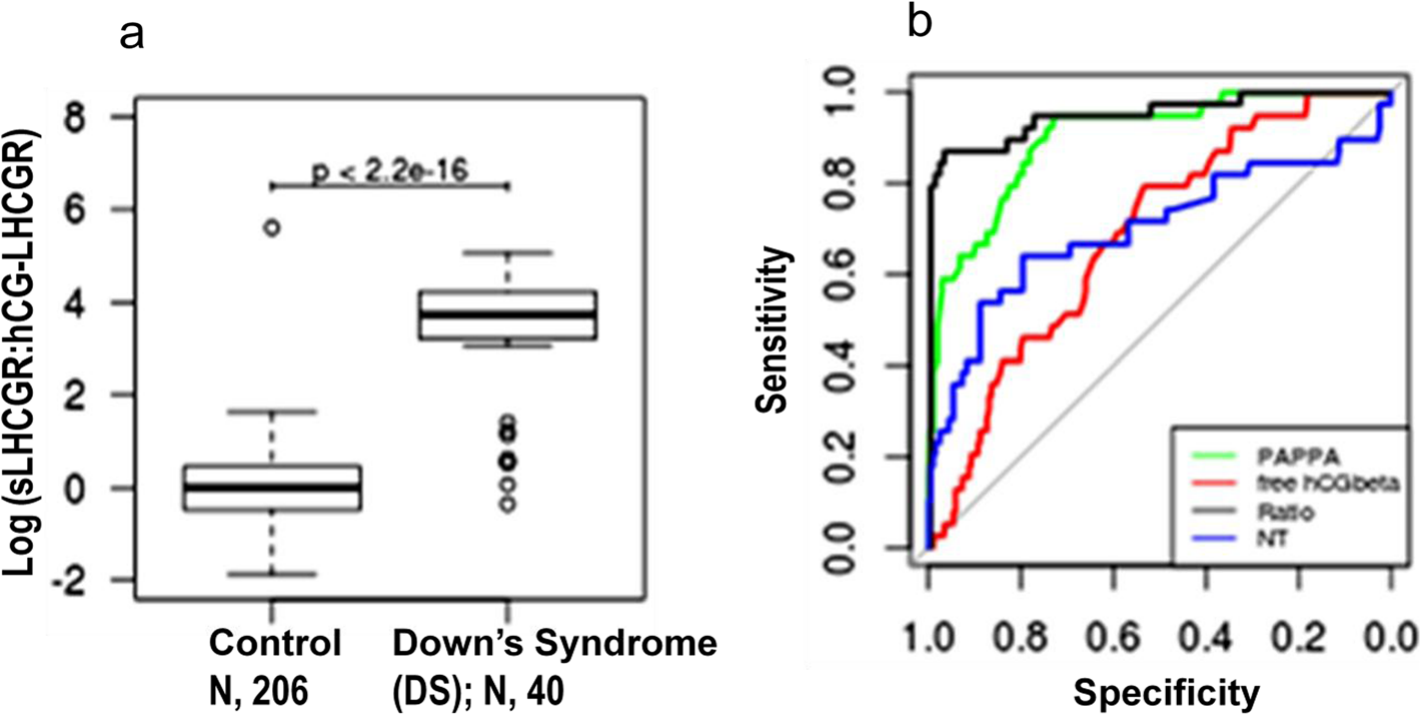
**The predictive value of the ratio of LHCGR forms in the detection of Down’s syndrome. a)** Box-plots showing sLHCGR MoM:hCG-sLHCGR MoM ratio of the control (N, 206) and DS (N, 40) pregnancies; **b)** The ROC for each Down’s marker. The ‘Ratio’ represents Log (sLHCGR:hCG-LHCGR).

300 control sample concentrations obtained for PAPP-A, free βhCG and NT were converted into multiples of the median (MoMs) for the corresponding gestational age and, after applying correction factors for diabetes, weight, IVF, ethnicity and smoking status, the risk at term for Down Syndrome, Edwards Syndrome and Patau’s Syndrome was estimated. Risk calculation was based on the Lifecycle program version 3.2 (Perkin Elmer). For diagnosis, the CVS (89.1%) or amniocentesis (10.9%) was programmed on the same day if the estimated risk for aneuploidies at term was 1:250 or above. The sLHCGR and hCG-LHCGR ELISA assays were carried out exactly as described [8] except with the following modifications: 50 μl of 5 to 10-fold diluted serum was incubated in antibody-coated plates for 15 min prior to adding 100 μl of Horse Radish Peroxidase-labelled detection antibody for another 90 min. Following six washes, the plates were further incubated with 100 μl of TMB for 15–30 min. Following measurement of each analyte, the ratio of the multiplicity of median (MoM) sLHCGR :MoM hCG-sLHCGR was calculated for each serum.To visualize the difference in the distribution of results in DS cases and controls, the data were log-transformed and plotted in a box-plot (see Figure 1a). A small quantity, equal to 1/10 of the smallest measurable sample concentration, was artificially added to the hCG-sLHCGR values to avoid zeros in the log-transformation. The statistical significance of the difference in the medians of the two samples was calculated using a Wilcoxon rank-sum method.

The median Trisomy 21 DR and 95% confidence intervals at 5% and 10% FPR were determined by re-sampling using the R pROC package. AUC_DF_ is the estimated area under the smoothed receiver operating characteristic (ROC) curve which was calculated using a distribution-free method [9]. Smoothing was accomplished by the default method in the pROC package or by the 'density' method of the same package as appropriate. The ROC curves for each Down’s marker including sLHCGR/hCG-LHCGR ratio were determined using pROC as described [10].

## Results

Consistent with our published data [8], the serum hCG-sLHCGR together with PAPP-A detected additional DS pregnancies which were negative by free hCGbeta plus PAPP-A screening procedure (discussed below). Therefore, sLHCGR/hCG-sLHCGR has an additive effect on the current primary biochemical screening of aneuploid pregnancies at the first trimester.

In an alternative analysis of 40 Down’s and 300 control samples, the sensitivity of multiple biochemical markers combined with NT at FPR of 5% and 10% were calculated. The results are shown in Table 1. It is evident that, in a two-marker combination, PAPP-A plus hCG-sLHCGR provided the highest DR of 81% and 84% at 5% and 10% FPR, respectively. Moreover, addition of the third marker (NT) in combination with PAPP-A + hCG-sLHCGR reduced the FPR from 10% to 5% without affecting the DR (84%). Interestingly, the addition of a fourth marker (βhCG) which increased the DR to 90% had also increased the FPR to 10%. Of note, the maternal age and other parameters were not included in the algorithm.

**Table 1:** Detection of Trisomy 21 with fixed false positive rates

**Markers**	**AUC**_**DF**_	**5% FPR**	**10%FPR**
βhCG + PAPPA	0.918	0.49(0.27 – 0.73)	0.73(0.55 – 0.92)
PAPPA + NT	0.922	0.66(0.5 – 0.81)	0.78(0.64 – 0.9)
PAPPA + hCG-sLHCGR	0.92	0.81(0.68 – 0.91)	0.84(0.74 – 0.95)
βhCG + hCG-sLHCGR	0.856	0.53(0.39 – 0.75)	0.65(0.5 – 0.83)
βhCG + NT	0.753	0.23(0.11 – 0.37)	0.36(0.23 – 0.51)
hCG-sLHCGR + NT	0.888	0.71(0.56 – 0.84)	0.77(0.63 – 0.88)
NT + PAPPA + βhCG	0.94	0.59(0.35 – 0.83)	0.83(0.66 – 0.97)
NT + PAPPA + hCG-sLHCGR	0.928	0.84(0.72 – 0.95)	0.87(0.76 – 0.98)
hCG-sLHCGR + NT + PAPPA + βhCG	0.966	0.83(0.73 – 0.92)	0.9(0.82 – 0.97)

The data presented in Table 1, as well our previously published report [8], suggested that hCG-sLHCGR and sLHCGR had utility in combination with PAPP-A, free βhCG and other markers, but might be limited in use as independent markers. However, when the ratio of sLHCGR and hCG-sLHCGR was examined, it was found to be significantly higher in DS pregnancies compared to controls. Such analysis revealed that the sLHCGR forms (sLHCGR and hCG-sLHCGR ratio with a cut-off of ≥ 2.0) could detect 80% of Down’s cases with a FPR of 0.48% (Figure 1a).The results shown in Figure 1a prompted examination of the impact of individual biochemical markers including sLHCGR/hCG-sLHCGR ratio and NT on the screening of trisomy 21 pregnancies. The highest detection rates with an AUC of 0.9524 (0.9091-0.9958) for LHCGR/hCG-sLHCGR were 87.5% (75% - 97.5%) and 87.5% (77.5% - 97.5%) at FPR of 5% and 10%, respectively (Figure 1b). These results led to the conclusion that sLHCGR forms could act as independent markers in first trimester screening for Down’s syndrome and, as such, are more efficient than PAPP-A, free-βhCG or NT alone.

## Discussion

An important objective of this study was to examine whether serum LHCGR could increase the screening efficiency of PAPP-A plus free--βhCG which are currently used as first trimester biochemical markers together with NT for prenatal risk assessment of fetal aneuploidy. In order to compare the relative screening efficiencies in this study, first the DR and FPR at fixed cut-off values for PAPP-A (≤0.5 MoM), βhCG (≥1.7 MoM), hCG-sLHCGR (≤2.0 & ≥ 20.0 MoM) were calculated. Out of all combinations, the DR for Down’s syndrome with PAPP-A plus hCG-sLHCGR was highest (57.5%) with FPR of 2.3%. These results were comparable (DR, 58.1% and FPR, 4.5%) to our published data on 43 Down’s samples from two sources [8]. The additive effect of hCG-sLHCGR on PAPP-A and βhCG measurement was 35%, compared to 21% in published data, [8]. The additive effect is defined as T21 pregnancies identified by the PAPP-A + hCG-sLHCGR which could not be detected by conventional PAPP-A and βhCG measurements. Additionally, hCG-sLHCGR, in combination with PAPP-A, βhCG and NT (≥.2.0 MoM), detected 95% (38/40) DS pregnancies. Together, the data presented here demonstrate that serum sLHCGR/hCG-sLHCGR alone or in combination with existing markers can increase the DR and reduce the FPR in Down’s screening.A limitation of this study is that the cut-off value for sLHCGR:hCG-LHCGR ratio of ≥ 2.0 was established on analysis of samples derived from a single center (Figure 1). Therefore, it must be emphasized that larger population-based future studies, involving both low and high-risk groups, are required to establish the cut-off values for serum sLHCG/hCG-sLHCGR to detect DS.

Currently, the routine non-invasive prenatal testing (NIPT) involves the biochemical screening of maternal serum biomarkers (PAPP-A and free - βhCG) at 9–14 wks of gestation as well the measurement of fetal NT. The algorithms based on these results, and other parameters including maternal age, body mass index (BMI), parity (twin or singleton) etc., are used to assess the risk for fetal aneuploidy. The biochemical and NT testing together could detect 79-90% of trisomy 21 at a FPR of 5% [1,2]. The screen-positive pregnancies, following initial risk assessment, are referred to more invasive genetic and molecular testing by CVS/amniocentesis for definitive diagnosis of aneuploidy or other chromosomal abnormalities.

Current guidelines on NIPT proposed by the International Society for Prenatal Diagnosis and others [11-13] support the idea of cfDNA testing on ‘high-risk’ pregnancies only. The high-risk was defined on the basis of maternal age (≥35 yrs), screen-positives by biochemical and ultrasound testing, history of aneuploidy and parental balanced Robertsonian translocation associated with trisomy 13 and 21 [11]. In the absence of sufficient validation of the cfDNA testing for fetal aneuploidy, it should be considered as ‘advanced screening test’ and is not fully diagnostic. Additionally, the current cfDNA testing for aneuploidy is insufficient to account for half the chromosomal abnormalities detected by molecular analysis of samples derived from CVS or amniocentesis [14].

While non-invasive fetal DNA sequencing provides the highest sensitivity for T21 screening, a major disadvantage is the cost of introducing fetal DNA sequencing as a universal screening method for low as well as high risk populations. As discussed recently [15,16], a cost-effective prenatal screening strategy would initially employ the traditional first trimester screening and subsequently analyze only the high-risk pregnancies using the relatively expensive DNA-sequencing method. Therefore, increased sensitivity with simultaneous reduction in false positive rates in conventional prenatal screening could be the most economical avenue for successfully implementing the fetal DNA sequencing scheme for the diagnosis of trisomic 21 pregnancies in the general population.

Compared to the fetal DNA sequencing scheme, which is highly specific and sensitive [4,5,15,16], sLHCGR biomarkers are more broad-ranging. The hormone hCG is the earliest embryonic signal which directly modulates placental growth, angiogenesis and fetal development. Our current and previous analyses of >1000 pregnancies suggest that hCG functions are partly regulated by circulating sLHCGR. The serum sLHCGR, at intermediate concentrations appears to be necessary for maintaining normal pregnancy. However, soluble LHCGR at very low and extremely high concentrations are strongly associated with adverse pregnancy outcome. The dramatic reduction of full-length LHCGR expression in Down’s syndrome chorionic villi [6,7] points toward a physiological role for sLHCGR in hCG signalling at early pregnancy. While the precise nature of this role is not known, the impact of high serum LHCGR in pregnancy could be two-fold: reduced hCG bioactivity and aberrant systemic vasculo-endothelial and immune activation.

Given the fundamental role of hormone hCG and its receptor LHCGR throughout pregnancy, it is perhaps to be expected that measurement of sLHCGR forms may also detect other pregnancy pathologies, in addition to being a useful first trimester biochemical adjunct for the detection of DS. Indeed, sLHCGR forms have shown preliminary success as predictive diagnostics for a wide range of pregnancy pathologies including miscarriage, preeclampsia and pre-term delivery. The sLHCGR system may therefore complement, rather than directly compete with, existing and emerging technologies by providing early indication of those at most risk. It should be noted however that the diagnostic capacity of serum sLHCGR forms as biomarkers for other pregnancy pathologies, has yet to be extensively explored and requires full clinical validation.

## Conclusions

The free beta subunit of the pregnancy hormone (free- βhCG) together with PAPP-A constitutes the fundamental serum analytes for prenatal screening of Down’s and other trisomic fetuses at the first trimester of pregnancy. Previous studies showed that soluble hCG receptor (sLHCGR) in combination with PAPP-A could detect Down’s pregnancies which remained undetected by PAPP-A plus free- βhCG. The present study suggests that the ratio of the serum LHCGR forms (sLHCGR:hCG-sLHCGR) could serve as a superior independent marker for Down’s syndrome and significantly increase the efficiency of the current prenatal screening program without compromising the false positive rate.

## Competing interests

The use of sLHCGR-based immunodiagnostic tests has been patented by Origin Biomarkers.

## Authors’ contributions

LRB, AM, LL and MP participated in designing the study in co-ordination with SB, EC, FC and EG; AEC, IM and EC together performed the ELISAs, EC provided the PAPP-A, free βhCG and NT MoM values and clinical diagnoses based on ultrasound, biochemical (PAPP-A and hCGβ) and biometric analyses; EC, AEC, WEM together with SB analyzed the data, contributed to the interpretation of results, creation of manuscript figures and revised drafts of the manuscript; WEM performed statistical analysis on the data; SB created the first draft of the manuscript. All authors read and approved the final manuscript.

## Pre-publication history

The pre-publication history for this paper can be accessed here:

http://www.biomedcentral.com/1471-2393/14/197/prepub
